# Ultrasound-Guided Synovial Biopsy: A Review

**DOI:** 10.3389/fmed.2021.632224

**Published:** 2021-04-22

**Authors:** Fernando Saraiva

**Affiliations:** ^1^Serviço de Reumatologia, Hospital de Santa Maria, Centro Hospitalar e Universitário de Lisboa Norte, Lisbon, Portugal; ^2^Unidade de Investigação em Reumatologia, Faculdade de Medicina, Universidade de Lisboa, Lisbon, Portugal

**Keywords:** ultrasound-guided synovial biopsy, synovial membrane, synovium, synovial tissue analysis, musculoskeletal ultrasound

## Abstract

Ultrasound-guided synovial biopsy is a safe, well-tolerated, and effective method to collect good-quality synovial tissue from all types of joints for clinical and research purposes. Although synovial biopsy cannot be used to distinguish between types of inflammatory rheumatic disease, analysis of synovial tissue has led to remarkable advances in the understanding of the pathobiology of rheumatoid arthritis and other inflammatory rheumatic diseases. Synovitis is the hallmark of these diseases; hence, accessing the core of the pathological process, synovial tissue, provides an opportunity to gather information with potential diagnostic and prognostic utility.

## Introduction

This review aims to gather, in a single source, the most comprehensive information on ultrasound-guided synovial biopsy (USGSB), including the unmet needs of the method.

USGSB is a method for retrieving synovial membrane samples using ultrasonography for guidance. The main advantages of USGSB are that it is well-tolerated by patients; is accessible; can feasibly be performed in large and small joints, as well as bursae and tendon sheaths; and has a low incidence of adverse events. USGSB uses ultrasound to visualize the location of synovial hypertrophy and is simple to perform after adequate training, suitable for serial procedures, and comparable to arthroscopy (which is considered the gold standard for obtaining synovial samples, based on clinical trial data) in terms of sample quality, but is less invasive, is cheaper, and does not require ionizing radiation, unlike fluoroscopy-guided biopsies (1–5). The main disadvantages of USGSB, relative to arthroscopic biopsy, are the limited tissue quantity obtained and lack of direct vision. Moreover, when using USGSB, the synovial tissue retrieved depends on the degree of synovitis and requires musculoskeletal and ultrasound guidance skills. Major contraindications to USGSB are systemic or skin infection, coagulation disorders, or anticoagulant therapy, as well as a non-collaborating patient.

In this review, we address the following questions: Why are synovial biopsies conducted? How are synovial biopsies conducted? What data do synovial biopsies provide? and What are the unmet needs related to synovial biopsy?

## Why Are Synovial Biopsies Conducted?

**Figure 1:**
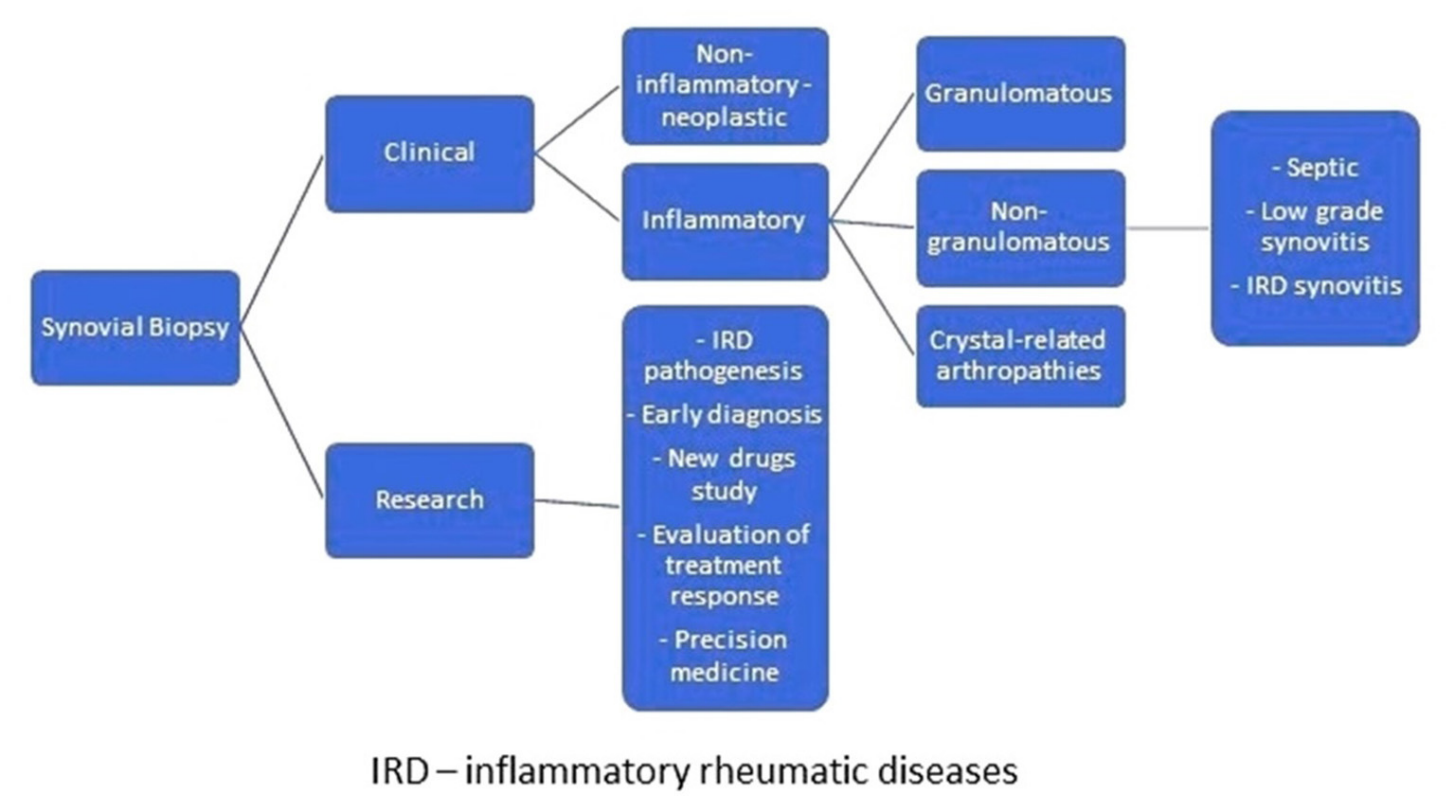
Why are synovial biopsies conducted?

Synovial biopsies are conducted for clinical reasons or research purposes (6, 7). In the clinical setting, formal indication for synovial biopsy occurs in cases of monoarthritis for which all other auxiliary diagnostic tests, including synovial fluid examination, are insufficient for diagnosis. Clinical biopsies are performed to exclude infection or because diagnostic clarification is required; for example, to identify whether synovitis has a non-inflammatory or inflammatory cause. Tumors cause non-inflammatory synovitis, while crystal-related arthropathies and granulomatous and non-granulomatous diseases are responsible for inflammatory synovitis. Crystal-related arthropathies are due to sodium monourate, calcium pyrophosphate, or basic calcium phosphate deposition diseases. Granulomatous synovitis can be related to infection (tuberculosis, brucellosis, fungal infections), immunological (Crohn's disease, erythema nodosum, sarcoidosis), metabolic, and storage diseases, or to a reaction to a foreign body. Non-granulomatous synovitis may be due to septic arthritis, low-grade synovitis (post-traumatic, mechanical, osteoarthritis, or haemochromatosis-related synovitis), or high-grade synovitis (rheumatic inflammatory diseases, such as diffuse connective tissue diseases or spondyloarthritis) (Figure 1). Bacterial and fungal diseases can be identified by detection of broad-range 16S and 18S ribosomal RNA in synovial tissue by polymerase chain reaction, which is valuable for the diagnosis of infection by these agents (4).

Synovial biopsies are conducted for research purposes in the context of the study of inflammatory rheumatic disease pathogenesis, early diagnosis, new drugs (through recognition of novel therapeutic targets), identification of biomarkers of disease progression, or advance precision medicine (8, 9). Nevertheless, synovial biopsy is not yet sufficiently discriminative to allow distinction between types of arthritis (10).

## How are Synovial Biopsies Conducted?

### Comparison of Synovial Biopsy Approaches

**Figure 2:**
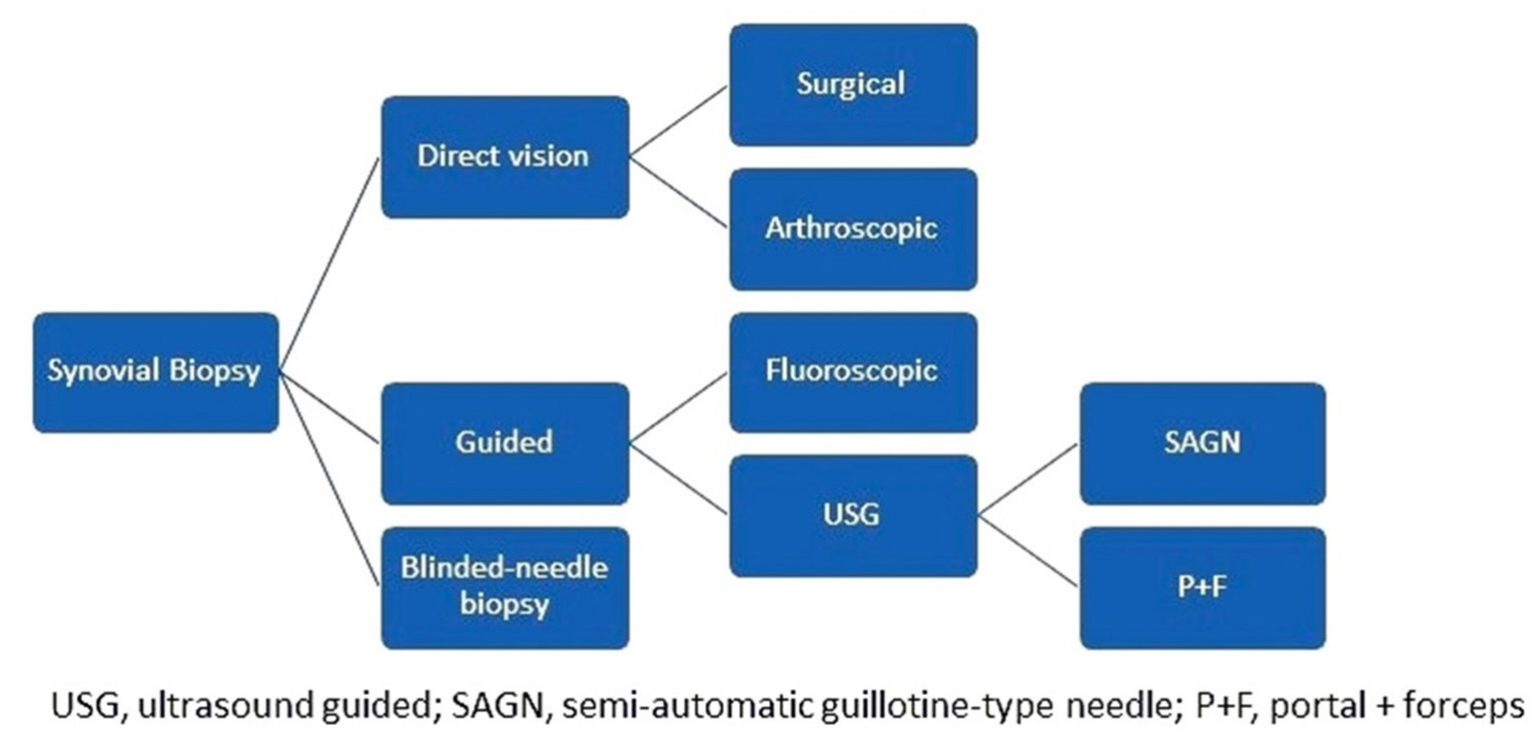
How are synovial biopsies conducted?

Synovial biopsies can be achieved in the context of surgery (arthrotomies, arthroplasties) fluoroscopic or arthroscopic guided, or conducted as a blind needle- or ultrasound-guided procedure. USGSB is conducted as either a portal + forceps biopsy (PFB) or a guillotine-type semiautomatic needle biopsy (NB). PFB can be rigid or flexible, while NB can be conducted with or without a coaxial; coaxial is helpful when there is a long pathway to deep-seated joints, or when the needle passes near neurovascular structures (Figure 2). USGSB clearly has the most well-balanced profile among synovial biopsy approaches, when cost, technical simplicity, patient acceptability, synovial sampling success rate, suitability for large and small joints, and suitability for serial biopsies are considered (2). Of interest, synovial tissue quality is maintained and clinical and ultrasound evaluations do not change when repeat USGSB is necessary for the same joint (2, 7, 11, 12). Blind needle- and fluoroscopy-guided biopsies are more difficult to conduct in small joints and joints without active synovitis and have a lower success rate for obtaining synovial samples than other methods (2, 5). Cost and technical simplicity favor blind needle biopsy, while suitability for serial biopsies and biopsies in small joints favor USGSB, particularly NB (2, 13–15). The major differences between the two types of USGSB are that PFB requires an autoclave for equipment sterilization, one guide, larger ports, and dual operators and is more time consuming (10), while NB uses disposable material, can be easily achieved by a single operator, and is most appropriate for finger, toe, and wrist joints (the joints most frequently affected in rheumatoid arthritis); however, the choice between PFB and NB depends primarily on operator preference and experience.

Compared with arthroscopic biopsy, USGSB is less invasive and can be conducted in both large and small joints (6). Arthroscopic biopsy allows direct visualization of synovial membranes but is more costly, is performed in only a few specialized centers, requires two operators and a dedicated environment (an operating theater or equivalent), and is not viable for small joints (1, 2, 13, 14).

There are no differences between arthroscopic biopsy, PFB, and NB in terms of synovial sample quality, adverse events, or reported patient outcomes. Also, synovial pathotype and degree of inflammatory infiltrate are not influenced by the method used to retrieve synovial samples (2, 3).

Synovitis grade (synovial thickness) on pre-biopsy grayscale ultrasound scan is the best predictor of successful USGSB, in terms of synovial sampling and grading. Kelly et al. proposed a joint selection hierarchy for biopsy, based on joint size and synovitis grade on grayscale ultrasound (16). According to this hierarchy, the first choice should be a medium or large joint, with grade 3 synovial thickening, followed by a joint of any size with synovitis grade ≥ 2, a medium or large joint with synovitis grade ≥ 1, and finally, a small joint with any degree of synovitis. Although not a contraindication, performing a synovial biopsy in a grade 1 synovitis is technically harder to accomplish, especially in the hands of a non-experienced operator.

### The USGSB Procedure

In any rheumatological center, several steps should be implemented to adequately perform USGSB. In the author's department, the first step is a checklist, which includes patient reception; exclusion of procedure contraindications; written informed consent; clinical data collection, including biometric items, tender joint count, swollen joint count, erythrocyte sedimentation rate, and C-reactive protein levels; patient- and physician-scored global activity (visual analog scale; VAS); ongoing treatment; and factors related to the joint to be biopsied, including VAS scores for pain, stiffness, and swollenness in the previous week.

The biopsy procedure should be carried out in a theater or clean procedure room, providing sufficient expanse for a sterile trolley, a bed, and an ultrasound machine. Most USGSBs are best performed with the patient in the supine position and the physician seated. Before the procedure, a brief ultrasound examination of the joint to be biopsied is performed to plan the most adequate needle path, in order to avoid tendinous and neurovascular structures, and synovitis grade is determined, according to grayscale and power Doppler.

**Figure 3:**
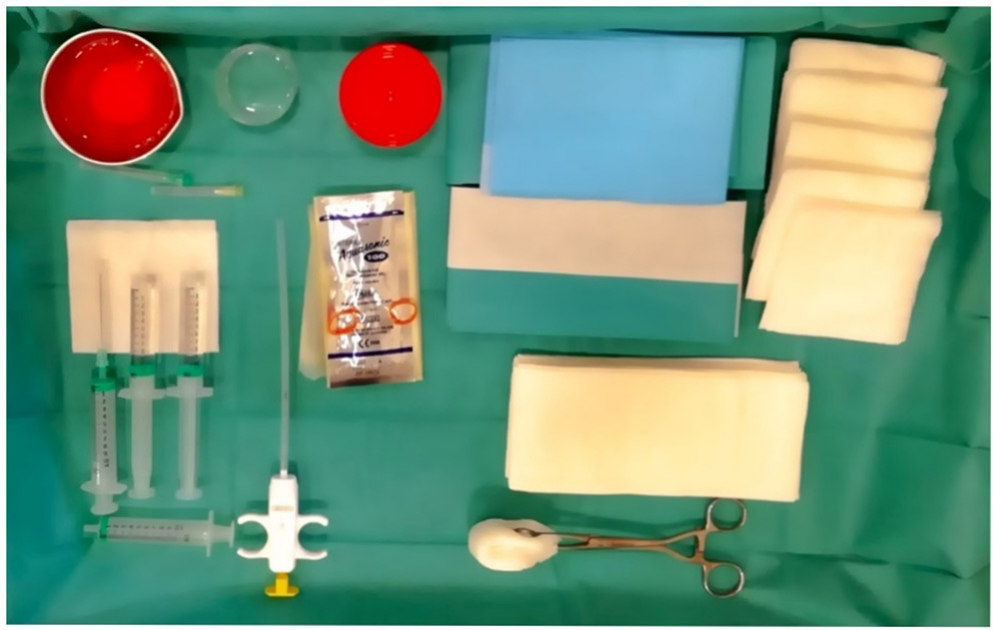
Materials required for USGSB.

**Figure 4:**
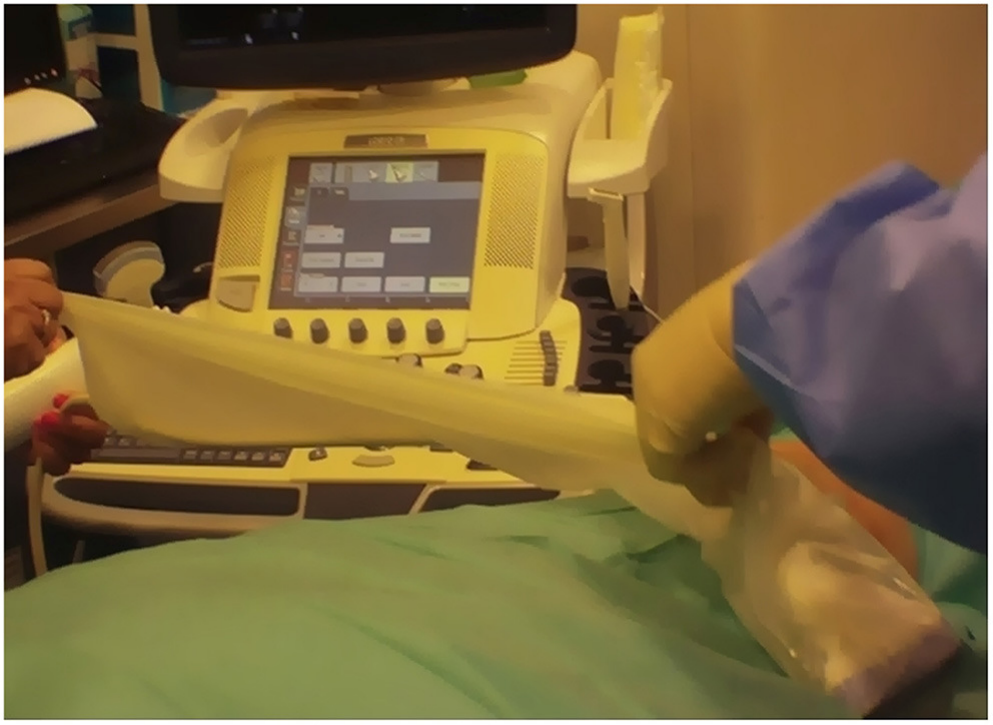
Field and probe cable protection.

**Figure 5:**
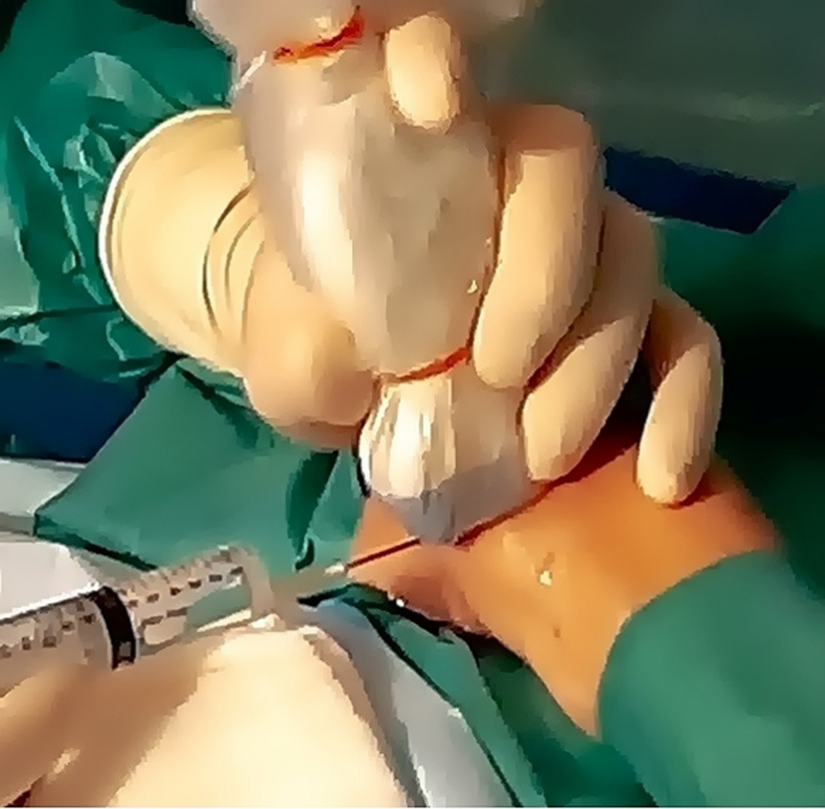
Local anesthesia.

**Figure 6:**
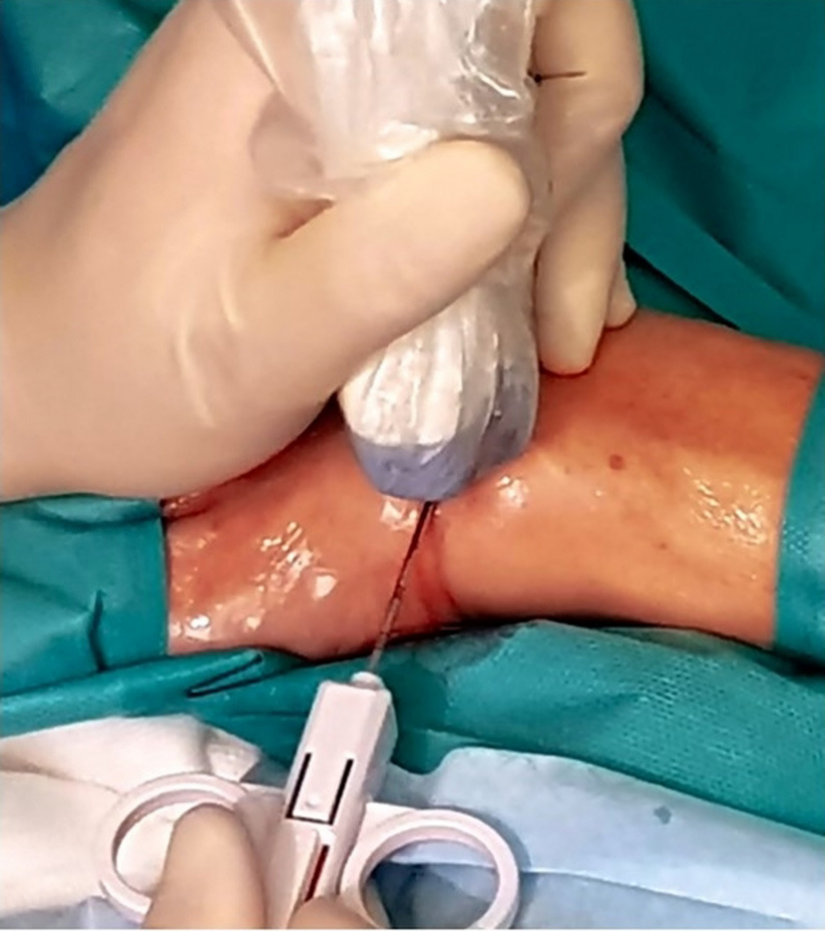
Insertion of biopsy needle.

**Figure 7:**
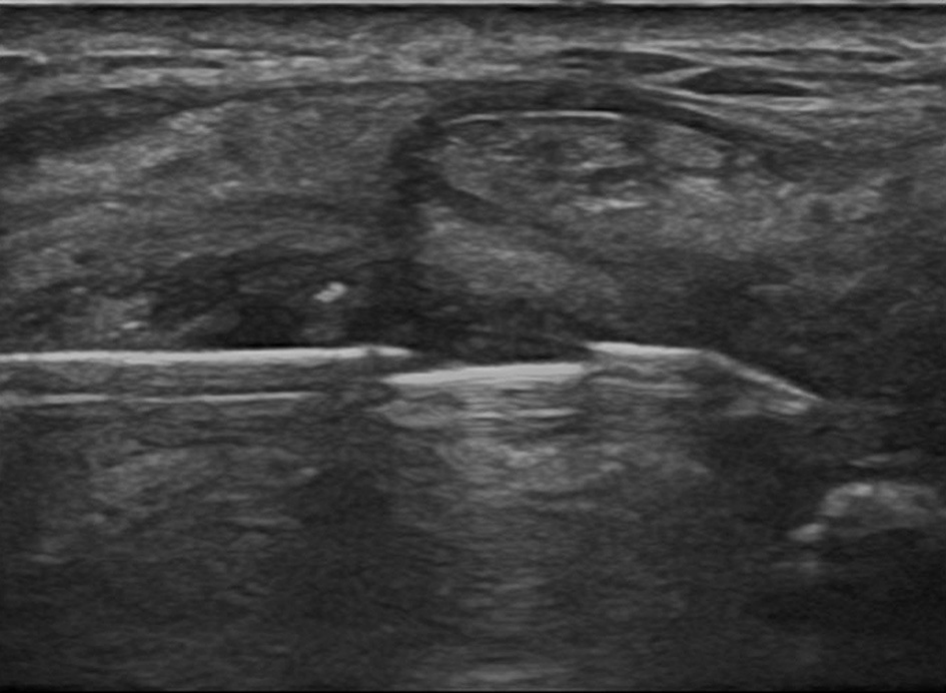
Biopsy needle inside the joint.

**Figure 8:**
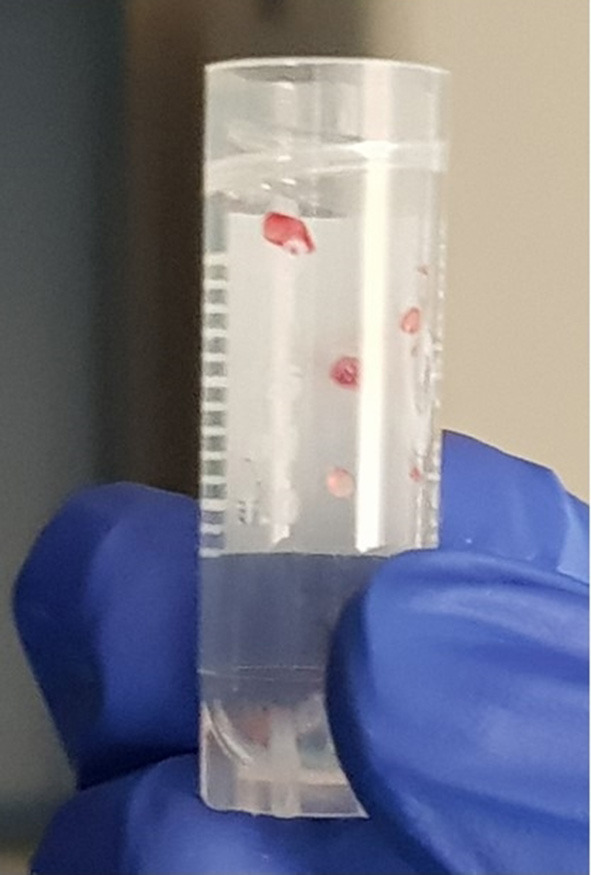
Synovial samples.

A support table, with all required equipment and materials, is prepared and a sterile technique implemented. Sterile gel or chlorhexidine solution must be used as a contact medium. An in-plane approach, trying to keep the needle as parallel as possible to the probe, is the best option. Then, each USGSB procedure follows a similar routine with fluid aspirated (if present) and 1–5 ml of local anesthetic injected into the soft tissues up to the joint capsule, under US guidance. A further 2–5 ml of 1% lidocaine is instilled into small joints, 10–15 ml for large joints. For large joints such the knee, hip, or shoulder, a suitable coaxial outer needle may be used in addition to the 14- or 16-G biopsy needle, to facilitate repeated needle entry over a long path. For small and intermediate joints, a 16-G, throw-length 10-mm biopsy needle is used, without a coaxial sheath. US imaging is used to guide the needle to an appropriate predetermined biopsy site. After the procedure, compression of the entry site is followed by the application of a small adhesive dressing. Some of the steps involved are illustrated in Figures 3–8.

When the procedure is completed, the patient is asked to grade his/her tolerance for the intervention. In our experience with NB, the time spent for the whole procedure is <70 min, of which more than half is spent in the pre-procedure steps, with the biopsy itself usually lasting <35 min. Pre-procedure steps include the checklist (around 10 min); brief ultrasound examination (5 min); and operator, material, and patient preparation (20 min = 5 + 10 + 5 min, respectively). Five to 14 days post-biopsy, patients are asked to grade their discomfort during the procedure and whether they took over the counter analgesics. Besides, they are requested to grade the pain, stiffness, and swollenness in the biopsied joint over the previous 3–4 days and their willingness to repeat the procedure, if needed. Any adverse events that may have occurred are recorded and ultrasound examination of the biopsied joint performed.

### Synovial Sample Collection and Processing

Synovial sample collection is driven by the procedure goal—clinical or research. For clinical purposes, ≥6 fragments are sent for paraffin embedding and ≥5 for microbiology. Fragments sent for paraffin embedding are then sectioned into thin slices using a microtome and sent for histopathological analysis, following staining with hematoxylin–eosin. For research purposes, ≥6 fragments are sent for paraffin or optimal cutting temperature cryoprotective compound embedding. After thin sections are cut, samples are analyzed by immunohistochemistry or immunofluorescence for cell-type identification, according to the research goal. We also store ≥ 6 fragments in RNALater, which is a substance that counteracts RNA degradation by RNAases, for subsequent transcriptome analysis (7). The final number of samples collected inevitably depends on and reflects the research goal, patient tolerability, and time available (10).

The European Synovitis Study Group (ESSG) recommends as quality criteria that biopsy size should be >2.5 mm^2^ and that the synovial lining layer, as well as the overall morphology of the tissue, must be preserved (17). The same group recommends that synovitis should be quantified; one of the most popular scoring systems for that purpose is Krenn's score, which assesses three features of synovitis: hyperplasia of the lining cell layer, stromal cell density, and intensity of inflammatory infiltrate. Krenn's score can range from 0 to 9, as follows: 0–1, no; 2–4, low-grade; and 5–9, high-grade synovitis (18). The ESSG also recommends that the synovial pathotype, as well as the presence of ectopic lymphoid follicles, should be described (17). Other authors have recommended six as the minimum number of samples to collect for immunohistochemistry, to reduce T cell variability; however, the minimum number of samples for other cell types is less well-established. For macrophages, it depends on the goals pursued by the research (6). Other measures of synovial tissue quality include the number or percentage of synovial fragments and gradable synovial fragments, the total area of gradable synovial tissue, the area of gradable tissue by biopsy sample, and the RNA integrity number (RIN) (2); RIN ranges from 1 to 10, where samples from synovial tissue with values > 3 are considered adequate for subsequent transcriptomic analysis (2), molecular analysis, gene expression profiling, or gene sequencing methods (e.g., next-generation sequencing) (5). The capacity of USGSB to retrieve sufficient RNA is remarkable, even from small joints with minimal synovitis.

## What Data Do Synovial Biopsies Provide?

### Healthy Synovial Membrane

Synovial tissue analysis is a fundamental tool for investigating arthritis pathobiology and searching for biomarkers of treatment response in basic and translational research, and is a useful method in routine clinical practice (11). To better understand the value of USGSB, it is crucial to have adequate knowledge of the “normal” synovial membrane or synovium, which is an ectoderm-derived structure that coats the inner surface of diarthrodial joints. The synovium has folds or villi, which provide non-restrictive motion and an augmented absorptive area; may be discontinuous, resulting in occasional bare areas of cartilage or bone; and contains two parts, the intimal lining layer, which is in contact with the joint cavity, and the stroma or sublining layer, with no basal lamina or membrane between them. The microanatomy of the normal synovial membrane can be divided into three types, fibrous, areolar, and adipose, according to the structure and contents of the sublining layer, with the areolar subtype the most typical and ubiquitous, the adipose subtype primarily found in fat pads, and the fibrous subtype found in finger and toe joints (19).

The normal synovial membrane has several functions: (1) it allows the movement of adjacent relatively non-deformable structures; (2) it maintains an intact non-adherent tissue surface; (3) it controls synovial fluid volume and composition; (4) it lubricates cartilage; (5) it nourishes chondrocytes; and (6) it absorbs debris and metabolic waste products (19, 20).

Normal intima is formed by one or two layers of synoviocytes. These can be (1) type A synoviocytes, which are macrophage-like and derived from blood monocytes via subintimal venules, or (2) type B synoviocytes, which are the most abundant, locally derived fibroblast-like synoviocytes of mesenchymal origin. The usual pattern found in the lining layer is a first row of type A synoviocytes, in close contact with the joint cavity, below which there is a row of type B synoviocytes (19). Type A synoviocytes are capable of phagocytosis and pinocytosis and have numerous micro-filopodia and prominent Golgi apparatus. Type B synoviocytes have prominent rough endoplasmic reticulum organelles and synthesize hyaluronan, fibronectin, laminin, collagens, catabolin, lubricin, “superficial zone protein,” neutral proteinases, collagenase, and gelatinase. Type B synoviocytes also express several adhesion molecules, including vascular cell adhesion molecule 1 (VCAM-1), intercellular adhesion molecule-1 (ICAM-1), β1 integrins, and the surface markers, CD44 and CD55. The extracellular connective tissue matrix of the lining layer includes a fine fibrillary net of type I, III, IV, V, and VI collagens, as well as variable amounts of hyaluronan, laminin, fibronectin, chondroitin-6-sulfate-rich proteoglycan, and fibrillin-1 microfibrils (10, 19, 20). Large amounts of hyaluronan are present in the lining layer, as well as in the part of the sublining layer that is closer to the lining layer, while there is no hyaluronan deeper in the subintimal layer.

The normal sublining layer is relatively acellular and contains some blood and lymphatic vessels. Fibroblasts and adipocytes are the dominant cell types in the sublining layer, while a few macrophages, B lymphocytes, plasma cells, CD3^+^, CD4^+^, and CD8^+^ T lymphocytes, granzyme B-positive cells, interdigitating antigen-presenting dendritic cells, and mast cells may also be found, but in small numbers (19). The part of the sublining layer closer to the lining layer also has a row, two to three cells thick, beneath which there are capillaries, with a deeper plexus of small arterioles and venules, with lymphatic vessels found deepest (furthest from the lining layer) in the sublining layer (19). A rich network of sympathetic and sensory nerves is present in the synovium, usually associated with blood vessels, and extending into the intimal layer. These nerves are myelinated and terminate close to blood vessels, whose vascular tone they regulate. Sensory nerves respond to proprioception and pain via large myelinated nerve fibers or myelinated fibers with unmyelinated free endings (nociceptors), or via small unmyelinated fibers. Synovium nociceptors are reactive to neuropeptides, including vasoactive intestinal peptide, calcitonin gene-related peptide, and substance P (20). The sublining layer contains many elastic fibers, providing tautness to this part of the synovium, which is mainly composed of loose connective tissue, but contains other constituents, including laminin, fibronectin, chondroitin-6-sulfate-rich proteoglycans, and type I, III, IV, V, and VI collagens (19, 21).

Synovial macrophages are CD163^+^, CD68^+^, CD11b^+^, and CD14^+^; however, subintimal macrophages are strongly positive for CD14, while intimal macrophages are only weakly positive. There are other differences between markers expressed by intimal and subintimal macrophages in healthy synovium: intimal macrophages express strong non-specific esterase (NSE) activity and the immunoglobulin receptor, FcgRIIIa, while subintimal macrophages are weakly positive for NSE activity and express the immunoglobulin receptor, FcgRI, with low or absent levels of FcgRIIIa (19, 21).

Regarding cytokine production in normal synovium, although very small amounts of pro-inflammatory cytokines, such as interleukin-1β (IL1β), interleukin 6 (IL-6), and tumor necrosis factor-alpha (TNF) are generated, levels are much lower than those present in any type of synovitis. In contrast, levels of IL1β receptor antagonist and osteoprotegerin, which inhibits receptor activator of NF kappa B ligand (RANKL), in the normal synovium far exceed those of the molecules they inhibit (19).

### Synovial Pathology

In the context of clinical arthritis, analysis of synovial tissue allows the identification of lymphocytic aggregates that can produce local autoantibodies, potential biomarkers differentiating rheumatoid arthritis (RA) from other forms of early arthritis, distinct fibroblast cell types in involved or non-involved arthritic joints, and decreased macrophage numbers as a surrogate marker of treatment response (1, 13). Indeed, decreased number of macrophages (CD45^+^ or CD68^+^) in the synovial sublining is the most reproducible and validated biomarker of treatment efficacy in RA, and more reliable than disease activity score 28 (DAS28) (12, 13, 22). In contrast, macrophage infiltrate density correlates with progressive structural damage. In addition to macrophages, B cell aggregates and mast cells are also associated with severe disease in RA (23–28). Other potential biomarkers in synovial tissue include different populations of lymphocytes and lymphocyte aggregates, cytokines, chemokines, S100 proteins, adhesion molecules, mediators, and degradation products from bone, cartilage, and synovial membrane, various antigens and antibodies, and genes involved in the regulation of cell division and immune responses (9). The degree of infiltration and aggregation of lymphocytes in synovial tissue, the number of CD68^+^ macrophages in the sublining, and global synovial inflammation scores, as well as levels of pro-inflammatory cytokines, chemokines, and catabolic enzymes in the synovium, all correlate with disease activity, erosive burden, and radiographic progression. Flow cytometry, bulk-RNA sequencing, and single-cell technologies (mass cytometry and single-cell RNA sequencing), used in judicious sequence, have facilitated high-resolution identification of disease-associated cell subsets in human tissues. These technologies have enabled the identification of four distinct fibroblast populations in the synovium (three in the sublining layer and one in the lining layer); four populations of monocytes; six of T cells (three CD4^+^ and three CD8^+^); and four of B cells (naïve B cells, memory B cells, autoimmune-associated B cells, and plasmablasts) (29). Taken together, these data demonstrate that analysis of synovial tissue is an important tool with prognostic value, in terms of phenotypic variability, disease activity, and disease severity (21).

The Doppler signal in RA correlates with hyperplasia of the synovial lining, lymphoid and macrophage infiltration of the sublining, angiogenesis, and lympho-myeloid pathotype (9, 30–32). Both the lining and sublining layers of the synovium exhibit typical features during rheumatoid synovitis. In the lining layer, rheumatoid synovitis manifests as hyperplasia, due to synoviocyte proliferation, with a dramatic increase of type A synoviocytes, which may account for up to 80% of the intimal layer, and mononuclear cell infiltration, caused by recruitment of bone marrow-derived monocytes, reaching up to 12 cells in thickness; these two types of cells are important sources of cytokines, chemokines, matrix-degrading enzymes, adhesion molecules, and osteoclastic and angiogenic factors. Further, increased fibronectin content, with resultant fibrin deposition in the superficial layer of the inflamed synovium and expansion of the lining layer and increased metabolic demands, is observed, but with few blood vessels in the vicinity, suggesting relative hypoxia, a potent stimulus to produce VEGF and other angiogenic mediators.

In the sublining layer, rheumatoid synovitis causes stromal proliferation, with pronounced infiltration by monocytes, macrophages (mainly pro-inflammatory M1 macrophages, monocyte-derived), T cells (mainly, but not limited to, CD4^+^ Th1 and Th17, and CD45 RO^+^ T cells), B cells and plasma cells (mainly antibody-producing, but also chemokine and cytokine producers), dendritic cells (myeloid and plasmacytoid dendritic cells, which are antigen-presenting cells, producers of inflammatory mediators and cytokines, and possibly also involved in local autoantibody production), NK cells (a source of IL-22, and mediator of fibroblast proliferation), mast cells (antigen-presenting cells, osteoclast promoters, cytokine, and histamine producers), and neutrophils (source of citrullinated peptides, proteases, cytokines, and reactive oxygen species). Metalloproteinases are also increased, alongside stimulators of osteoclast activity, enhanced angiogenesis (with immature blood vessels, expression of adhesion molecules in the vascular endothelium, and formation of numerous endothelial venules), lymphatic congestion, formation of ectopic lymphoid structures (lymphoid aggregates with germinal centers), granulation tissue, fibrin deposition, and fibrinoid necrosis in the sublining layer (8, 10, 19, 21, 30, 33, 34).

### Classification of Synovitis Based on Immune Cell Analysis

Immunohistochemistry and immunofluorescence analyses in early arthritis, to identify the dominant cells and the inflammatory infiltrate intensity, allow classification of different pathotypes, namely, the three synovial phenotypes: lympho-myeloid, diffuse-myeloid, and pauci-immune/fibroid, each one affecting about a third of patients (32, 35, 36). Cell types analyzed include CD3^+^ (T cells), CD15^+^ (neutrophils), CD20^+^ CD22^+^ (B cells), CD21^+^ (dendritic follicular cells), CD31^+^ and F VIII (endothelial vascular cells), CD38^+^ CD138^+^ (plasma cells), CD45^+^ CD68^+^ CD163^+^ CD90^−^ (macrophages), CD90^+^ CD45^−^ CD55^+^ (fibroblasts), and CD117^+^ (mast cells). Biopsies are stratified for each of these groups by semiquantitative grading (0–4) of immunohistochemistry results revealing the degree of immune cell infiltration, which is classified as follows: lympho-myeloid, CD20^+^ cells ≥ 2 and/or CD138^+^ cells ≥ 2; diffuse myeloid, sublining layer CD68^+^ cells ≥ 2, CD20^+^ cells ≤ 1, and/or CD3^+^ cells ≥ 1, and CD138^+^ cells ≤ 2; and pauci-immune/fibroid, sublining layer CD68^+^ cells <2 and CD3^+^, CD20^+^, and CD 138^+^ cells <1 (18, 32).

The lympho-myeloid pathotype is characterized by the dominance of B cells and plasma cells, together with myeloid cells, and can be associated with autoantibodies, osteoclast-related genes, disease activity, structural damage, poor response to csDMARDs, and good response to anti-IL 6 drugs. The hallmark of the pauci-immune/fibroid pathotype is stroma-resident cells, in association with scarce immune cells, low levels of autoantibodies, lower activity, and less damage/disease progression, but poor response to treatment. The myeloid-diffuse pathotype is characterized by the presence of myeloid cells and scarce B cells, has intermediate features, relative to the two other phenotypes, and may respond well to anti-TNF drugs (32, 35). It has been postulated that, as the pauci-immune pathotype occurs in a considerable proportion of cases of early arthritis, this is a defined pathotype, rather than representing a burned-out end-stage disease (35). Moreover, Lliso-Ribera et al. (36) showed that synovial pathotypes can distinguish between clinical phenotypes, independently of disease duration. Together, these data question the dogma of the “opportunity window” in RA treatment and demonstrate that patients with RA that fulfill the 1987 ACR criteria have an increased probability of needing biologic disease-modifying anti-rheumatic drugs (bDMARDs) at some point during the disease course, relative to those with undifferentiated arthritis or those that fulfill the 2010 ACR/EULAR criteria, but not the 1987 ACR criteria (36). Patients needing bDMARDs exhibit upregulation of genes encoding factors mediating the proliferation, differentiation, and activation of B and T cells, and of matrix metallopeptidase production and cytokine-mediated cell activation. Patients who do not require bDMARDs mainly express genes regulating fibroblast proliferation and cartilage turnover. Finally, the integration of histologic and molecular signatures improves the sensitivity and specificity of a model for predicting which patients would need bDMARDs at some point during the disease course (36).

Regarding established RA, immunohistochemistry and immunofluorescence can also identify several pathotypes, whose various designations include diffuse, aggregate, lymphoid, granulomatous, follicular, myeloid, fibroblastic, and pauci-immune/fibroid synovitis (8, 37–39). Diffuse or myeloid synovitis affects 50–70% of patients, is associated with good responses to anti-TNF drugs, and has one of the most benign clinical phenotypes, where rheumatoid factors tend to be absent, CD68^+^ cells predominate, and there are few lymphocytes and no ectopic lymphoid structures (39–41). Aggregate, follicular, or lymphoid synovitis is associated with more active disease and the presence of rheumatoid factor and tends to respond to anti-IL6 drugs. It affects 22–50% of patients and may comprise two subtypes, one associated with follicular dendritic cell networks or ectopic/tertiary lymphoid-like structures, and allegedly with worse outcome, and another which lacks those networks/features (37–40). In follicular or lymphoid synovitis, B cell infiltrates and B cell markers predominate, while pauci-immune or fibroid synovitis is characterized by a fibroblast-rich landscape, overexpression of cellular and molecular markers of macrophages and fibroblasts, almost no immune cell infiltration, no associated rheumatoid factor, and a poor response to anti-TNF or anti-IL6 drugs (8, 39); this subtype affects 20–30% of patients. Another much rarer pathotype, granulomatous synovitis, affects <1% of patients and is associated with extra-articular features (38). Fibroblast-like synoviocytes with distinct genetic signatures are also associated with different disease phenotypes and outcomes (40); however, conflicting results have been reported regarding the association of synovial lymphoid aggregates and disease severity (41–44). Nevertheless, the overall positive correlation between lymphoid aggregates and synovial inflammation may simply be the result of interdependency, rather than mutual exclusivity, between lymphoid and myeloid infiltrates (41). It should also be stressed that synovitis scores and pathotypes may vary among samples from the same biopsy, because of minor differences between sections of the same sample. Hence, final results should be defined only after consideration of several samples and sections (37).

The existence of such diversified pathotypes indicates the presence of distinct pathogenic pathways in the synovial membrane and the need for therapeutic strategies directed toward every scenario (17); however, some authors have proposed that these data provide evidence of two main pathogenic pathways, one through a lymphoid axis, targeted by drugs like tocilizumab, rituximab, and methotrexate, and another through a myeloid axis, targeted by anti-TNF drugs (41).

### Clinical Utility of Synovial Biopsies

#### Synovial Biopsy to Distinguish Synovitis Pathotypes

In a study conducted in the author's department, the median synovitis score was significantly superior in follicular pathotype RA (5.8 ± 0.0), relative to the diffuse (4.0 ± 1.3) and fibroid (2.5 ± 0.5) pathotypes. In the same study, the median synovitis score was comparable between different inflammatory rheumatic diseases but considerably superior to that in primarily non-inflammatory conditions, such as osteoarthritis, synovial chondromatosis, or foreign body synovitis (37). Comparison of pathotypes among different rheumatic inflammatory conditions did not reveal significant differences between various diagnoses, except for a clear predominance of the diffuse pathotype in spondylarthritis (SpA), the absence of the fibroid pathotype in crystal-related arthropathies, and absence of the follicular pathotype in primarily non-inflammatory arthritis (37).

Synovial membrane activation of JUN N-terminal kinase is increased in early RA, but not in undifferentiated forms of arthritis, and CD22^+^ and CD38^+^ cells distinguish RA from other forms of arthritis (9). Compared with psoriatic arthritis (PsA), RA synovial membranes contain fewer vessels, lower lipid content, and fewer neutrophils, mast cells, CD163^+^, and CD117^+^ cells, with equal levels of lining and sublining CD68^+^ cells, sublining CD3^+^, CD20^+^, and CD21^+^ cells, but higher levels of CD138^+^ cells, and these differences appear to be independent of therapy (26, 45, 46). DMARD-naïve PsA patients that had reached minimum disease activity (MDA) at 6 months had lower levels of CD3^+^ cells than those that did not reach MDA at 6 months. Similarly, naïve patients with RA that reached DAS28 remission at 6 months had lower levels of CD68^+^ cells than those that did not reach remission at 6 months (45).

Patients with undifferentiated peripheral inflammatory arthritis that evolved to a definite diagnosis had higher levels of lining and sublining CD68^+^ and CD3^+^ cells than those who did not evolve to a definite diagnosis. Similarly, patients with higher grayscale ultrasound and power Doppler ultrasound scores were more likely to evolve to a definite diagnosis than those who did not (47).

Other features that may help to differentiate RA from SpA and other diagnoses are the higher ratios of CD3^+^/CD4^+^ T cells, RANKL/OPG, and CD20^+^/CD22^+^ B cells in the former. RA patients also have more cell infiltration (B and T cells), more lymphoid aggregates, and higher numbers of CD68^+^ sublining macrophages and CD38^+^ plasma cells than those with SpA, but lower M2 macrophage populations and, eventually, fewer blood vessels in the sublining layer (21, 27, 34, 48, 49).

#### Quality and Quantity of Synovial Samples Collected by USGSB

Humby et al. conducted a study in small joints of 35 patients with RA, to determine whether USGSB at baseline and at second biopsy could generate sufficient high-quality synovial tissue for pathotype identification, RNA extraction, and detection of sublining macrophage changes after treatment. They showed that good-quality synovial tissue, adequately reflecting synovial phenotype, was obtained in 81% of biopsies when synovial hypertrophy > 2 was detected by grayscale ultrasound pre-biopsy, opposed to 20% of those with a minimal degree of synovial hypertrophy. In all biopsies, it was possible to retrieve enough RNA for molecular analysis, regardless of pre-biopsy synovial hypertrophy grade, as determined by ultrasound, and a significant correlation was detected between the change in the number of sublining CD68^+^ macrophages and treatment response evaluated by DAS28. Moreover, they showed that it was necessary to examine multiple biopsy samples, and not only multiple sections of the same sample, to obtain a representative image of the cell infiltrate; use of at least four samples produced a good result, while examination of ≥10 samples (where possible) increased the percentage of evaluable tissue substantially, in terms of CD3^+^, CD20^+^, and CD68^+^ cellular infiltrates. Further, they demonstrated that, in patients with minimal synovial hypertrophy, increasing the number of samples did not improve the quantity of gradable synovial tissue, nor did the presence of Doppler signal predict the success of the procedure (22).

Kelly et al. performed 93 synovial biopsies in large, as well as small, joints, in patients with early RA, of which 36 had a second biopsy 6 months later. A median of 14 samples was retrieved per joint, and 93% of biopsies yielded good-quality synovial tissue, which was maintained in even the second biopsy. RNA samples extracted from all joints and all biopsies were adequate, even those from repeat biopsies, although gradable tissue was only obtained from 40% of small joints. The quantity and quality of synovial tissue retrieved correlated with elevated synovitis score determined by pre-biopsy ultrasound grayscale examination, but not with power-Doppler grade. Self-limited joint discomfort, solved in <24 h, was the most frequent adverse event, occurring in 19% of patients (16).

Published data show that USGSB facilitates the collection of synovial samples of sufficient quality in 82–96% of biopsies, compared with 48–85% of blind needle biopsies (4–6, 32, 37, 50–52). Results are also influenced by operator expertise, joint size and type, and synovitis grade, as determined by grayscale ultrasound (6). In our department, of a series of 64 NB performed in all kinds of joints, synovial bursae, and tendon sheaths, 81% were done within clinical practice to investigate a possible infection, or to help to clarify a diagnosis, and 19% in the context of research activities (37). Synovial biopsy had diagnostic and treatment impact in 37% of cases and, in the research setting, 92% of cases could be used for the proposed objectives. Of all biopsies, 88% yielded synovial tissue, consistent with literature reports (4, 37). Notably, the median sample number was significantly lower in biopsies from which synovium was not successfully retrieved and, of eight unsuccessful biopsies, six were from large joints. Operator experience also had a clear impact on the quality of synovial tissue obtained (37). Remarkably, synovial tissue was less likely to be successfully obtained from samples collected later in the procedure and had fewer concordant pathotypes than those collected early. Consequently, the authors recommend that samples collected for different purposes should be assigned in parallel, rather than sequentially (37).

#### Synovial Biopsy to Evaluate Disease Progression and Treatment Response

Alivernini et al. used ultrasound and synovial immunohistochemistry to study 42 patients with undifferentiated peripheral arthritis with rheumatoid factor, who were anti-citrullinated protein antibodies (ACPA) negative and naïve to DMARDs. They found a correlation between CD68^+^ lining and sublining layer cells and CD 31^+^ (intravascular) cells and ultrasound scores. The few patients that evolved to a definite diagnosis had significantly higher levels of the aforementioned cells and higher ultrasound scores and CD3^+^ cell numbers in the sublining layer. These patients also exhibited downregulation of miRNAs 346 and 214 (47).

Just et al. conducted baseline and 6-month ultrasound, magnetic resonance imaging (MRI), and synovial biopsy assessments in 20 patients with early RA and 20 with established RA. They found that EULAR-OMERACT ultrasound score and RAMRIS MRI score were strongly correlated with Krenn's synovitis score at baseline, but not 6-month assessment, in the early RA group. In the established RA group, a moderate to strong correlation was present between the three scores at baseline assessment, except for the RAMRIS 6-month assessment, which was not performed (53).

Rivellese et al. detected CD20^+^ cell infiltrate ≥ 2 (0–4) and B-cell-rich synovitis in 35% of DMARDs-naïve early RA and 47.7% of established RA, with inadequate responses to anti-TNF, which were significant differences. They also found that patients with B-cell-rich synovitis had higher levels of disease activity, rheumatoid factor, and ACPA positivity, but only in early RA, not established RA. Nevertheless, patients with both early and established RA with B-cell synovitis also had higher total histologic synovitis scores. According to the authors, this lack of correlation between B-cell-rich synovitis and clinical disease activity scores (DAS28) could be a sign of the insensitivity of clinical scores for capturing synovial inflammation and may explain the progressive structural damage in patients with low clinical disease activity (54).

Two independent groups compared ACPA-positive and -negative patients with established RA and found that synovial numbers of CD3^+^, CD8^+^, CD19^+^, and B cells were significantly higher in ACPA-positive patients; however, they also had more active disease, introducing bias in the analysis (55, 56).

After 3 months of treatment with tocilizumab, a marked decrease in CD3^+^ cells was detected in patients with early RA (57). After 3 months of treatment with adalimumab, patients with RA and an inadequate response to methotrexate had a marked decrease in CD 68^+^ cells (58). Finally, 3 months of treatment with rituximab resulted in a significant reduction in both B cells and IL-17-producing T cells in patients with RA (59).

The R4RA study investigated the best therapeutic option between rituximab vs. tocilizumab for patients with RA and previous inadequate response to anti-TNF, by assessing whether molecular and cellular signatures of B cells predicted a better response to rituximab. Tocilizumab showed better results in the B cell-poor group and was not inferior to rituximab in the B cell-rich group, although there was a higher incidence of adverse events (60).

#### Diagnostic Value of USGSB

In RA, there is a tendency to find similar numbers of cells in the sublining layers of different joints in the same patient, even between clinically involved and non-involved joints, although less pronounced in clinically uninvolved joints; however, this does not occur in the synovial lining layer, in which there are no similarities in the number or characteristics of macrophages and fibroblast-like synoviocytes between clinically involved and non-involved joints, with distinctive DNA fingerprints and methylation patterns, according to their positional memory (9).

In septic arthritis, compared with synovial fluid analysis, USGSB increased the percentage of causative agent isolation in non-specific infections, as well as mycobacterial infections (6). Perivascular infiltrate of neutrophils, which typically comprise > 20% of all cells in synovial tissue of septic arthritis, as well as polymerase chain reaction for identification of bacteria and fungi, have remarkably high sensitivity and specificity for detecting the presence of infection. In addition to infectious arthritis, there are several other situations in which synovial biopsy may be diagnostic, including Whipple and Wilson diseases, synovial chondromatosis, pigmented villonodular synovitis, ochronosis, synovial lipoma arborescens, foreign body synovitis, crystal-related arthropathies, amyloidosis, hemochromatosis, histiocytic and neoplastic diseases, and sarcoidosis (6).

### Adverse Events Related to USGSB

Adverse events are uncommon in USGSB and usually mild and transient; however, adverse event rates <0.5%, as described in some series, seem unrealistic and likely reflect a low-sensitivity data collection strategy (3, 5, 7). Reported adverse events include vasovagal reaction; sensory disturbance; nerve, vessel, tendon, ligament, or muscle lesion; ecchymosis, hemarthrosis, skin, or joint infection; sinus tract from joint to skin; fracture of biopsy needle; and thrombophlebitis or deep venous thrombosis (11, 16, 22, 61). Transient post-procedure sensory disturbance, vasovagal reaction (1–2% of cases) and joint discomfort post-biopsy (usually <24 h; 7–19% of cases) are the most reported adverse events (5, 10, 16, 37). In a series of 524 synovial biopsies (402 NB, 65 PFB, and 57 arthroscopic), adverse events were detected in 1.5% of procedures, with no difference among methods. All patients reported clear improvement 2 weeks post-procedure. Repeated biopsies did not increase the number of adverse events or patient-reported outcomes (3). In our series, adverse events were considered discreet and transient, except for a single case of a slight limitation of fifth finger extension, which persisted after a wrist biopsy (37).

## Discussion

### What Are the Unmet Needs Related to Synovial Biopsy?

Our capacity to induce sustained remission or cure of RA and other inflammatory arthritis at the individual level remains limited, due to insufficient information to drive treatment. As synovitis is the hallmark of these diseases, accessing the core site of the pathological process (synovial tissue) provides opportunities to gather information with potential diagnostic and prognostic utility. Synovial tissue biomarkers appear an attractive target for that purpose, due to the inadequacy of peripheral blood biomarkers. An equivalent path has been followed in oncology, where histopathology has demonstrated prognostic value and is now integrated into the standard of care (15).

Several unmet needs related to USGSB and synovial tissue analysis remain a challenge for those working in the field of rheumatology, as outlined in Box 1. The ultimate goal is to find the ideal treatment, in the right time frame for each patient, using a precision medicine approach, as applied in cancer therapy. We believe that analysis of synovial tissue will play a decisive role in this strategy, hopefully in the near future (5, 15, 40).

Box 1 **Unmet needs related to USGSB and synovial tissue analysis.**

**General Framework:**

More robust validation in multiple centers

Compare the procedure, performance, and safety of PFB and NB appropriately, to produce a consensual definition of a successful procedureDefine training goals for practitioners learning how to perform USGSB

**Specific Technical Issues:**

Standardize sample acquisition (number of samples according to goals, define sites within the joint), processing, analysis, and reporting of results Pathotype:

- How to manage dynamic changes in pathotype that occur over the disease course and after treatment
- How many observations are required to define the dominant pathotype, since it may change according to the harvesting sequence, across samples from the same joint, and across joints from the same patient

Define the best cutoff to differentiate pathotypes that share overlapping features.

**Main Technical Goals:**

Identification of synovial biomarkers that distinguish between different types of arthritis and other diseases

Determine if synovial histopathological heterogeneity translates into diverse clinical phenotypes

Determine if it is an adequate method for patient stratification and treatment monitoring

Search for molecular signatures modulated by specific therapeutic approaches

Determine whether synovial biopsies early in the disease course can predict outcomes and identify patients that will respond to bDMARDs/csDMARDs and those who will not

The absence of robust, predictive biomarkers of treatment outcomes is a major unmet need in the management of RA and other types of inflammatory arthritis. A precise understanding of the key events occurring during synovitis will be critical in advancing the era of precision medicine in RA and other inflammatory rheumatic diseases, placing synovial tissue analysis at the core of this journey. However, before proposing routine use of standardized synovial tissue biopsy to guide therapy, several factors must be satisfied, according to the OMERACT Synovial Tissue Biopsy Special Interest Group, including uniformity of biopsy handling and analysis, validation of quality scores, and relationship between immunopathology and therapeutic response and between disease pathotypes and outcomes (62).

### Conclusion

In conclusion, available data demonstrate that USGSB is an effective, safe, and well-tolerated method of retrieving quality synovial tissue from any type of joint, with impacts on diagnosis and treatment. In the clinical setting, formal indication for synovial biopsy occurs mainly in monoarthritis cases, to exclude infection, and although synovial biopsy still cannot be used to distinguish between types of inflammatory rheumatic diseases, it has led to remarkable advances in the understanding of the pathobiology of RA and other inflammatory rheumatic diseases. Histopathological analysis, immunohistochemistry, and omic and molecular analyses of synovial tissue have brought us to the cusp of an era of personalized medicine in rheumatology.

## Author Contributions

FS is responsible for the conception of the manuscript, including the review of the literature and the individual experience that conducted to the idea.

## Conflict of Interest

The author declares that the research was conducted in the absence of any commercial or financial relationships that could be construed as a potential conflict of interest.
